# Zygote arrest 1 gene in pig, cattle and human: evidence of different transcript variants in male and female germ cells

**DOI:** 10.1186/1477-7827-4-12

**Published:** 2006-03-21

**Authors:** Svetlana Uzbekova, Monica Roy-Sabau, Rozenn Dalbiès-Tran, Christine Perreau, Pascal Papillier, Florence Mompart, Aurore Thelie, Sophie Pennetier, Juliette Cognie, Veronique Cadoret, Dominique Royere, Philippe Monget, Pascal Mermillod

**Affiliations:** 1 Physiologie de la Reproduction et des Comportements, UMR 6175 Institut National de la Recherche Agronomique/Centre National de la Recherche Scientifique, Université François Rabelais de Tours, Haras Nationaux, 37380 Nouzilly, France; 2 Service de Médecine et Biologie de la Reproduction, UMR 6175, Centre Hospitalier Universitaire Bretonneau, 37044 Tours, France; 3 Laboratoire de Génétique Cellulaire, INRA, Chemin de Borde-Rouge – Auzeville, BP 52627 31326 Castanet-Tolosan Cedex, France

## Abstract

**Background:**

Zygote arrest 1 (ZAR1) is one of the few known oocyte-specific maternal-effect genes essential for the beginning of embryo development discovered in mice. This gene is evolutionary conserved in vertebrates and ZAR1 protein is characterized by the presence of atypical plant homeobox zing finger domain, suggesting its role in transcription regulation. This work was aimed at the study of this gene, which could be one of the key regulators of successful preimplantation development of domestic animals, in pig and cattle, as compared with human.

**Methods:**

Screenings of somatic cell hybrid panels and in silico research were performed to characterize ZAR1 chromosome localization and sequences. Rapid amplification of cDNA ends was used to obtain full-length cDNAs. Spatio-temporal mRNA expression patterns were studied using Northern blot, reverse transcription coupled to polymerase chain reaction and in situ hybridization.

**Results:**

We demonstrated that ZAR1 is a single copy gene, positioned on chromosome 8 in pig and 6 in cattle, and several variants of correspondent cDNA were cloned from oocytes. Sequence analysis of ZAR1 cDNAs evidenced numerous short inverted repeats within the coding sequences and putative Pumilio-binding and embryo-deadenylation elements within the 3'-untranslated regions, indicating the potential regulation ways. We showed that ZAR1 expressed exclusively in oocytes in pig ovary, persisted during first cleavages in embryos developed in vivo and declined sharply in morulae and blastocysts. ZAR1 mRNA was also detected in testis, and, at lower level, in hypothalamus and pituitary in both species. For the first time, ZAR1 was localized in testicular germ cells, notably in round spermatids. In addition, in pig, cattle and human only shorter ZAR1 transcript variants resulting from alternative splicing were found in testis as compared to oocyte.

**Conclusion:**

Our data suggest that in addition to its role in early embryo development highlighted by expression pattern of full-length transcript in oocytes and early embryos, ZAR1 could also be implicated in the regulation of meiosis and post meiotic differentiation of male and female germ cells through expression of shorter splicing variants. Species conservation of ZAR1 expression and regulation underlines the central role of this gene in early reproductive processes.

## Background

Maternal mRNAs, accumulated in oocyte, have a crucial role in the success of early embryo development, allowing the first cleavages to occur, before the activation of embryonic genome [1]. Amongst the mRNA stored in the growing oocyte are some oocyte-specific genes called maternal effect genes which may account for this early cleavage regulation [2]. Maternal Antigen That Embryos Require (*Mater *or *Nalp5*) [3], Zygote Arrest1 (*Zar1*) [4], *Stella *[5] and nucleoplasmin 2 (*Npm2*) [6] are examples of maternal-effect genes that have been discovered in mice. They express preferentially in oocyte and knock-out (KO) of these genes leads to the incapacity of embryo to develop beyond the first cleavages. Some naturally-occurred point mutations in oocyte-specific *BMP15 *and *GDF9 *genes have been shown to increase ovulation rate in heterozygous carriers and to induce sterility in homozygous sheep [7]. On the other hand, developmental block of in vitro produced embryos remains the major problem in assisted reproduction technologies of domestic animals, particularly in cattle and pig. Growing number of data indicate that the stage of embryonic genome major activation, which is different between species, is crucial for the success of pre-implantation embryo development [8]. To ensure this maternal-embryo transition (MET) of gene expression, oocytes should reach a sufficient level of developmental competence during oocyte differentiation and maturation [9,10] Maternally expressed genes are widely implicated in this process and some of them were reported to be associated with developmental competence [11,12]. In domestic species, MET occurs later as compared to rodent (8-16-Cell-stage in cattle vs. 2-Cell stage in mouse), leaving more time to allow the study of the fate of maternal messengers and of their action in regulating embryo cleavage. Therefore, studying the maternal genes, including oocyte-specific ones, in farm species appears as a valuable model for the study of the mechanisms that affect oocyte quality and its implication in the success of embryo development and survival. The creation of subtracted cDNA libraries allowed recently the identification of novel oocyte-specific transcripts in bovine [13], [14]. In addition, homologues of some maternal-effect germinal cells specific genes were recently cloned in bovine (MATER, BMP15, GDF9 [15]; NALP9, [16]) and porcine species (VASA, [17]). Zygote arrest 1 (*Zar1*) was described in mice as maternal-effect oocyte-specific gene encoding putative transcription activator/repressor with an atypical plant homeobox domain (PHD) [4]. PHD zinc finger domain has a C4HC3-type motif, and is widely distributed in eukaryotes, being found in many chromatins regulatory factors [18]. By in silico sequences analysis,*Zar1 *was shown to be evolutionary conserved in six vertebrate species including human, mice, rat, xenopus, zebrafish and fugu [19]. ZAR1 expression was reported to be restricted to oocyte in mice and to ovary and testis in human, while in frog a larger pattern of expression, including ovary, lung and muscle, but not testis, was shown. In cattle the published data were contradictory: in a previous study we cloned partial ZAR1 cDNA, detected transcripts in oocytes and testis and showed the decreasing of mRNA throughout early embryo development [15], while expression of 126 bp fragment, showing the similarity with ZAR1, has been reported to be expressed throughout pre-implantation embryos development, as well as in ovary, testis, heart and muscle [20]. Here we cloned full-length cDNA of ZAR1 orthologues in pig *Sus scrofa *and cattle *Bos taurus*, characterised corresponding genes and their expression profiles in comparison with human and with our previous work on bovine [15], paying particular attention to mRNA differential expression and cellular localisation in reproductive tissues.

## Methods

### Oocytes, embryos and other tissue collections

#### Pig and cattle

All procedures were approved by the Agricultural and Scientific Research Government Committees in accordance with the guidelines for Care and Use of Agricultural Animals in Agricultural Research and Teaching (approval A37801).

Porcine (*Sus scrofa) *and bovine (*Bos taurus) *ovaries were collected at slaughterhouse and cumulus-oocyte complexes (COC) were aspirated from visible antral follicles. Bovine COC were subjected to in vitro maturation (MIV) for 22 hours followed by in vitro fertilization and embryo development, then embryos at 1-Cell, 2-Cell, 4-Cell, 5 to 8-Cell, morula and blastocyst stages were collected as described [15]. Groups of immature oocytes at germinal vesicle stage (GV) and mature oocytes at methaphase II stage (MII) were denuded from cumulus cells by mechanical treatment. Cumulus cells were centrifuged, washed in PBS and stored.

Production of pig embryos *in vivo *was conducted at the experimental farm of INRA Nouzilly (France) as described [21]. Briefly, thirteen Large White hyperprolific gilts were superovulated and artificially inseminated (AI) with semen from adult Pietrain boars. Donors were slaughtered in the INRA local slaughterhouse at day 1 – 5 after AI. The genital tract was collected immediately after slaughter and before scalding of the gilts. The number of corpora lutea on the ovaries was recorded and both uterine horns were flushed with 40–100 ml of saline solution (0.9% w/v NaCl) containing 2% (v/v) new-born calf serum (NBCS, Bio Whittaker, France). Each day from 1 to 5 after AI, groups of ten embryos were collected from 2–3 gilts and were classified as 1-Cell, 2-Cell, 4-Cell, 5 to 8-Cell, morula and blastocyst stages.

Biopsies (0.5–2 g) from different porcine tissues (hypothalamus, heart, pituitary, intestine, liver, lung, muscle, spleen, oviduct and ovary) were collected at INRA local slaughterhouse from two 7-weeks infantile gilts weighting about 15 kg. Ovaries were also collected from 7-months old peri-pubertal gilts of about 120 kg weight. Biopsies of testis were collected from young (7 months) and adult (2.5 years) slaughtered boars. Bovine biopsies of hypothalamus, pituitary, ovary and testis were collected from calves and adult cows or bull in an experimental setting. Granulosa cells were isolated from antral follicles and stored in pellets.

All samples were frozen in liquid nitrogen and kept at -80°C before experiements.

#### Human

After informed patient consent was obtained, immature oocytes at GV stage were recovered as deemed unusable for intracytoplasmic sperm injection (ICSI) from patients involved in a therapeutic ICSI program as described [22]. Oocytes were then stored individually in 10 μl of 1× RQ1-DNAse reaction buffer supplemented with 1 unit of RNAsin (Promega, Charbonnieres, France) at -80°C. Total RNA from adult human testis was kindly provided by Dr. J-L. Dacheux (INRA, Nouzilly, France).

### RNA and cDNA preparation and analysis

#### Total RNA preparation

Total RNA was extracted from bovine and porcine oocytes, embryos and biopsies by using TriZol reagent following the manufacturer's instructions (Invitrogen, Cergy Pontoise, France). Except for oocytes and embryos, RNA concentration was deduced from optical density and RNA integrity was checked by electrophoresis on denaturing gel. RNA from human oocytes was extracted by three times freezing – thawing procedure which was the following: tubes containing a single oocyte were plunged in liquid nitrogen for 1 min and then thawed in a water bath for 1 min at 37°C. To avoid the contamination with genomic DNA, total RNA preparations from oocytes and embryos or 2 μg of RNA from somatic and gonadic tissues were incubated with 1 unit of RQ1 DNAse (Promega) for 15 min at 37°C following by heat inactivation as described in manufacturer's protocol.

#### Complementary DNA (cDNA) synthesis

Routinely, reverse transcription (RT) was performed on RNA amounts equivalent to 5 oocytes or embryos, and on 1 μg of RNA from tissue biopsies. cDNA was extended from oligo (dT)_15_VN (V = A, C or G, N = A, T, C, or G) primers during 1 hour at 37°C by mouse Moloney leukaemia virus reverse transcriptase (Invitrogen) as described in user manual.

To obtain human oocyte cDNA, RT was performed directly from a half of a single oocyte RNA preparation.

Full-length cDNA from 35 -100 oocytes and 100 ng of tissue RNA was obtained using Super SMART PCR cDNA Synthesis Kits (Ozyme, St Quentin en Yveline, France) following manufacturer's instructions.

#### Cloning of porcine and bovine ZAR1 cDNA variants and sequence analysis

5'-and 3' rapid-amplification of cDNA ends (RACE) PCR were performed on bovine immature oocytes RNA and on porcine testis RNA using SMART RACE cDNA Amplification Kit (Ozyme). For RACE, specific primers were used: S10 and AS3 for pig and S1 and AS5-AS7 for cattle. Primers sequences and positions are listed in Table 1. ZAR1 cDNA variants from porcine oocytes were obtained by RT-PCR using S5 and AS1 primers, and from bovine oocytes using S2 and AS11. PCR mix from Advantage PCR kit (Ozyme) was complemented with 4% of DMSO. PCR products were cloned into the pCRII dual promoter vector using the TA cloning kit (Invitrogen). Clones containing presumptive ZAR1 inserts were sequenced locally using ABI Prism sequencing apparatus (Perkin Elmer, Courtaboeuf, France) or by Macrogen company (Seoul, South Korea).

**Table 1:** List of sense (S) and antisense (AS) primers used for ZAR1 mRNA analysis.

		Coordinate relative to ZAR1 coding sequences and exon position			
primer	sequence (5'-3')GB acc:	*bovine *DQ231456	*porcine *DQ231444	*human *AY191416	exon

S1	ccagccgagcaaggagcg	Bt (813)			1
S2	tacactgatggctgccctg	Bt (-8)			1
S3	tataacccttaccgagtggagg	Bt (976)		Hs (1096)	3
S4	ctcgtaccggtacccataccc			Hs (60)	1
S5	acgaggtgctggacggttaca		Ss (17)		1
S6	cctgcgcttccagttcttaga	Bt (831)	Ss (840)	Hs (952)	1
S7	tgccgaacatgccagaag	Bt (955)			1
S8	aacccgttccgcgacgtat	Bt (319)			1
S9	tgtctcggtgcagtgctcgtt		Ss (342)		1
S10	aacccttatcgcgtggaggata		Ss (988)		3
S11	tgcccagtaaaacttcgcca		Ss(1045)		4
AS1	tttgaagctgaaagtgctgtcac	Bt (1143)	Ss (1152)	Hs (1263)	4
AS2	tcttcctcgccgactcctct		Ss (691)		1
AS3	agagggagaaaggagaagagca		Ss(1261)		4
AS4	gacagctttgtcaaatacagcc		Ss(1307)		4
AS5	acaaatcttgacggaggggcct	Bt (1090)			4
AS6	gaagctgaaagtgctatcacag	Bt (1140)			4
AS7	ggcgacgatctctcgccagcggtgtcc	Bt (803)			1
AS8	ataggcgtttgcctttgcatc	Bt (1117)	Ss (1124)		4
AS9	ctggaagcgcaggcgctcct	Bt (843)			1
AS10	tcacaggataggcgtttgc	Bt (1124)			4
AS11	ttcacagcgggacctcagtt	Bt (1203)			4

The sequences obtained were analyzed using the software package proposed by Infobiogen [23]. Alignments were performed using BLASTn [24] and Multalin [25]. Site ENSEMBLE [26] was used for genome assembly. Deduced protein sequences were analyzed through the NCBI software [27] and Interpro [28] websites. Conservation of the blocks of synteny between human, cattle and pig was verified on the Multispecies Comparative Table [29], accessible on-line [30]. Search for repeated sequences was performed using REPuter software [31] accessible on-line [32]. Sequences of porcine and bovine ZAR1 genes and cDNA variants were directly submitted to GenBank.

#### Northern blot

A quantity of 20 μg of porcine and bovine total RNA from brain (hypothalamus), pituitary, testis and ovary was loaded onto MOPS-formaldehyde 1% agarose gel, migrated and transferred onto Hybond N+ membrane (Amersham Biosciences, Orsay, France) as described in manufacturer's manual. Hybridizations were performed in Ultrahyb solution (Ambion, Cambridgeshire, UK) following instructions. Porcine and bovine ZAR1 specific probes were amplified from plasmid DNA using S6 and AS8 primers (Table 1) and radio-labeled with α [32P]dCTP (Perkin Elmer) by "Prime-a-gene" labeling system (Promega).

#### Virtual northern blot

Two μg of amplified full-length cDNA obtained using SMART cDNA synthesis kit (Ozyme) from porcine and bovine immature oocytes were separated on gel and transferred onto HybondN+ membrane. Hybridizations were performed as described above for Northern blot.

#### RT- PCR analysis of ZAR1 expression in oocytes, embryos and tissues

For analysis of ZAR1 expression in oocyte and 1-, 2-, 4-, 5–8-Cell, morula and blastocyst stage embryos, we used as template cDNA amounts equivalent to one oocyte or embryo. For other tissues (hypothalamus, heart, pituitary, intestine, liver, lung, muscle, spleen, oviduct, cumulus and granulosa cells, ovary and testis), 5% of the reverse transcription products were used. In negative control reactions, a pool of RNA equivalent to 1 oocyte/embryo or 125 ng of tissues RNA was directly subjected to PCR. RT -PCR with water instead of RNA was also used as negative control. As positive control of cDNA quality, the β-actin specific PCR was performed with all samples using 5'-gcgtgacatcaaggagaagc-3' and 5'-tggaaggtggacagggaggc-3' primers, raising 432 bp fragment. S10-AS3 and/or S6 -AS8 primers were used for ZAR1 expression analysis in pig and cattle respectively. PCR mix (Interchim, Montluçon, France) was complemented with 4% of DMSO to amplify ZAR1 fragments. Amplified products were gel migrated, documented and then transferred onto nylon Hybond N+ membrane. Hybridizations were performed as reported [15], using porcine and bovine ZAR1 specific probes as described for Northern blot.

#### Comparative RT- PCR analysis of ZAR1 messengers in pig, cattle and human oocytes and testis

Template cDNA equivalent to 1/20 of RT from one oocyte (0.05 of oocyte RNA equivalent) and to 50 ng of reversed transcribed testis RNA were used for PCR reactions. Two sets of primers, designed within different ZAR1 cDNA regions named *a, b, c*, were used for each species. Region *a *was situated at the beginning of the first exon, downstream the first AUG start codon, while regions *b *and *c *were located within second, the most conservative half of cDNA. In pig, *a-c *primer set was S5-AS1 (amplicon of 1135 nucleotides) and *b-c *was S10-AS3 (amplicon of 273 nucleotides). In bovine, S8-AS9 primers were used as *a-c *set (amplicon of 524 nucleotide) and S6-AS8 primers, raising a 286 nucleotides product, as *b-c *set. Human S4-AS1 (amplicon of 1203 nucleotides) and S6-AS1 (amplicon of 311 nucleotides) primers were used as *a-c *and *b-c *sets, respectively. Amplified products were transferred onto nylon Hybond N+ membrane. Hybridizations were then performed using α [32P]dCTP -labeled fragment spanning 17–1152 nucleotides of pig ZAR1 cDNA as a probe.

#### Real-Time PCR

Real-time PCR was performed using an MyiQ (Bio-Rad Laboratories, Marnes La Coquette, France). Reverse transcription reactions were performed on RNA from six groups of 10 bovine oocytes at immature germinal vesicle (GV) stage and six groups at metaphase II stage after 22 h of MIV. 1 pg of luciferase mRNA was added to each group of 10 oocytes before RNA extraction and was used as external standard. Reactions were performed in triplicate using real-time PCR kit provided with a SYBR Green fluorophore (Bio-Rad) according to the manufacturer instructions. ZAR1 primers S7-AS10 and luciferase specific 5' -tcattcttcgccaaaagcactctg-3'and 5'-agcccatatccttgtcgtatccc-3' primers were used. The relative abundance of target cDNA was calculated using the DDCT I-Cycler IQ software. Analysis of variance by permutation score and Kruskal-Wallis tests was performed for statistical analysis of data. Difference was considered significant at p < 0.05.

#### In situ hybridization (ISH)

Frozen and paraffin-embedded ovaries from 7-weeks-old swine and testis from young adult boar were serially sectioned (10 μm) to perform in situ hybridization experiments as described [33]. Briefly, porcine ZAR1 specific ^35^S-labeled cRNA sense and antisense probes were obtained by in vitro transcription from 1 μg of T7-SP6 flanked fragment spanning 840–1124 nucleotides of pig ZAR1 cDNA. Paraffin sections were cleaned with toluene, rehydrated through serial ethanol-water dilutions and then incubated 7 min in PBS containing 4 μg/ml of Proteinase K (Sigma, France) at 37°C. After overnight hybridization and serial washes, slides were coated with NTB emulsion (Eastman Kodak Comp) and exposed in dark during three weeks at 4°C. Histological determination of gonad structures was assessed by staining the slides with Haematoxyline.

### Porcine and bovine genomic DNA extraction and analysis

#### Southern blot hybridization of genomic DNA in porcine and bovine

Genomic DNA was prepared from lysate of 200 mg of porcine and bovine follicular cells by double phenol-chloroform extraction. 10 μg of DNA were digested separately by Pvu II, Hind III or PstI restriction enzymes, separated on a 0.9% agarose gel and then transferred onto HybondN+ membrane. Hybridization with a ZAR1 specific probe as above (see Northern blot) was performed at 65°C overnight in 0.5 M phosphate buffer pH 7.4, containing 1 mM EDTA, 7% SDS and 50 μg/ml of salmon sperm DNA and membranes were finally subjected to autoradiography.

#### Regional assignment of Sus scrofa ZAR1 gene

Regional assignment was achieved by PCR using porcine specific primers S11-AS4 on a pig/rodent somatic cell hybrid panel comprising 27 hybrid clones [34], [35]. PCR amplification results were scored on 2% agarose gels. Regional assignment in pigs was achieved through hybrid cell analysis by using the statistical rules as defined by Chevalet et al. [36]. The probability of the regional assignment, the error risks, and the number of discordant had been taken into account when estimating the reliability of the localization.

Three BAC clones containing pig ZAR1 gene were found in INRA swine BAC collection [37] by PCR screening and sequencing of two of them was performed by primer walking (Macrogen).

## Results

### Cloning and comparison of ZAR1 cDNA from porcine and bovine oocytes

Human 1275 nucleotides full-length zygote arrest 1 cDNA sequence [GenBank: AY191416] was used as a query to Blast Search in *Sus scrofa *sequence collections accessible through GenBank. Primers S5 and AS1 were designed according to ESTs BX926143.1 and CO954861, showing 84% and 91% identity with 1–113 nucleotides and 1069–1274 nucleotides of human ZAR1 sequence, respectively. RT-PCR was performed on full-length cDNA from pig immature oocytes. Resulting PCR products revealed several sequences of different lengths which were homologous to human ZAR1 and showed 100% identity in overlapping with above ESTs. Three ZAR1 cDNA variants named oo1, oo2, oo3, containing 1164, 804 and 471 nucleotides open reading frames (ORF), encoding respectively 385, 267 and 146 amino acids putative proteins were obtained [GenBank: DQ231444, DQ231447, DQ231446].

In bovine, the partial cDNA corresponding to ZAR1 was previously cloned from immature oocytes RNA in our laboratory [15]. Here we performed 3'- and 5'- RACE-PCR in order to determine the full-length ZAR1 transcripts expressed in oocyte. Sequences of resulting clones revealed to be homologous to human and porcine ZAR1, and showed a perfect identity with *Bos taurus *chromosome genomic contig NW_974644. By alignment and assembling of 3'-and 5'-RACE sequences, 1485 bp full-length bovine ZAR1 cDNA was determined, which includes 1155 bp ORF and encodes putative 387 amino acid protein [GenBank: DQ231456]. Using S2 -AS11 primers, flanking the ORF, we cloned also five shorter variants of bovine ZAR1 cDNA. As in pig, they either presented ORFs, encoding putative shorter 295 and 170 amino acid ZAR1 proteins [GenBank: DQ231454 and DQ231450], or ZAR1 relative sequence with premature stop codons [GenBank: DQ231451, DQ231452, DQ231453].

**Figure 1:**
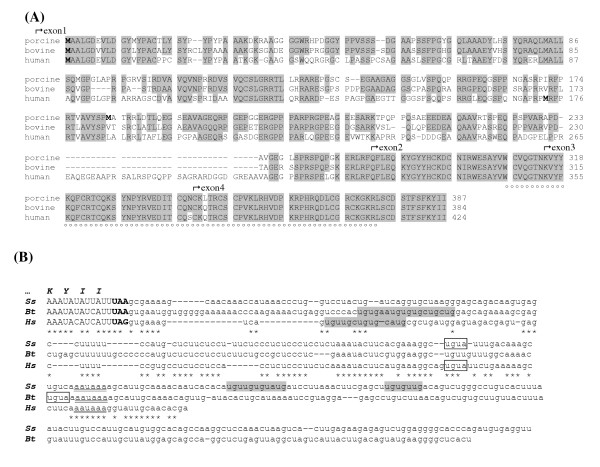
**ZAR1 protein and mRNA sequences comparison between pig, cattle and human. (A) **Alignment of porcine and bovine ZAR1 with human protein. Numbers indicate amino acid position. Identical amino acids are boxed in grey. Zinc finger, FYVE/PHD-type domain is underlined by circle points. Exon boundaries are defined by bent arrows. Start methionin amino acids (M) are in bold; note putative alternative start **M **sites in porcine (amino acid 183) and human (amino acid 173). **(B) **Porcine (***Ss***), bovine (***Bt***) and human (***Hs***) ZAR1 3'-- end cDNA alignment. 3-UTRs are in lower-case letters. Coding sequence is in capital letters. Termination codons are bold-typed. Identical nucleotides are denoted by stars. Protein sequence is in bold-type italic. Hexamer polyadenylation signals are double-underlined. Putative Pumilio-binding sites are outlined. U/purines dinucleotide-rich stretches (putative EDEN) are boxed in grey.

Comparison of ZAR1 coding sequences of *Sus scrofa *(Ss),*Bos taurus *(Bt) and *Homo sapiens *(Hs) showed a significant similarity (Fig. 1A). At the amino acid level, identity reached 78% between pig and cattle, while human ZAR1 showed 65% and 61% identity with porcine and bovine, respectively. Both pig and cattle proteins have the 36 amino acid gap comparing to human ZAR1. Highly conserved C-termini have 96% similarity between the species and contain FYVE/PHD zinc finger (ZnF) domain at 308–373 amino acid position in bovine and at 311–376 amino acid in porcine deduced proteins. At nucleotide level, porcine and bovine sequences revealed in totality 83% identity, showing significantly higher 93% homology within ZnF coding sequence. Numerous palindrome repeats were detected inside of GC-reach region spreading the first half of coding sequence in both species (Table 2). Within 3'- untranslated regions (3'-UTR), XPum (Pumilio) recognition sites UGUA, approximate to hexamer polyadenylation signals (HPS) AAUAAA, as well as sequences rich in U/(G/A) dinucleotides were found (Fig. 1B). Different length of 3'-UTR in bovine oocyte was evidenced by 3'-RACE [GenBank: DQ231448 and DQ231449]. Poly-A tail was found 197 or 323 bp after stop codon. In pig, RACE allowed to determine three closely positioned poly-A binding sites at 158, 167 and 207 nucleotides down-stream UAA.

**Table 2:** The longest palindrome repeats* in porcine and bovine ZAR1 coding sequences.

Palindrome repeats length (nt)	Starting positions of repeats relative to coding ZAR1 region		Calculated e-value of repeat**
***Sus scrofa***			
15	135	475	1.60e-02
12	77	102	8.18e-01
12	79	202	8.18e-01
12	268	584	8.18e-01
10	269	605	3.63e-01
12	539	789	8.18e-01
10	266	463	3.63e-01
***Bos Taurus***			
18	203	423	7.52e-03
15	41	451	3.30e-01
15	75	212	3.30e-01
13	206	631	2.18e-01
11	137	751	8.95e-02
10	117	560	3.58e-01
10	137	277	3.58e-01
10	267	636	3.58e-01

* Hamming/edit distance of repeats, computed by REPuter software, was ≤ 1.

**Calculated e-value of repeats – number of repeats of the same length or longer and with the same number of errors or fewer that one could expect to find in a random DNA of the same length.

### ZAR1 gene characterization and mapping in pig and cattle

We determined ZAR1 gene sequences and structure for both pig and cattle [GenBank: DQ231443 and DQ231455]. Without promoter region, genes spanned about 4 kb, and include 4 exons and 3 introns. Their structure was very similar to human ZAR1 gene AY191416 (Table 3), as well to mice *Zar1 *[GenBank: AY193889].

**Table 3:** Exon-intron structure of ZAR1 genes in pig and cattle comparing with human.

	***Sus scrofa***			***Bos taurus***			***Homo sapiens***		
exon/intron	coordinates relative to coding sequence	exon length (nt)	intron length (nt)	coordinates relative to coding sequence	exon length (nt)	intron length (nt)	coordinates relative to coding sequence	exon length (nt)	intron length (nt)

1	1–852	>852	1466	1–850	>850	1467	1–963	>963	1512
2	853–945	92	82	851–943	92	83	964–1057	92	80
3	946–1020	74	1292	944–1018	74	1099	1058–1132	74	1089
4	1021- 1164	>143		1019- 1155	>136		1133–1275	>142	

* Hamming/edit distance of repeats, computed by REPuter software, was ≤ 1. **Calculated e-value of repeats – number of repeats of the same length or longer and with the same number of errors or fewer that one could expect to find in a random DNA of the same length.

Mapping of pig ZAR1 by screening somatic cell hybrid panel allowed its assignment to chromosome 8 region 1/2p21-p23 with a regional probability of 0.82 and an error risk inferior to 0.1. This mapping result was totally in accordance with previous comparative mapping data available between human and pig. According to multispecies table of genes synteny, we found that human 4p11 and porcine 8 1/2p21-p23 locus, bearing ZAR1, correspond to bovine 6 chromosome region. More detailed analysis by screening of bovine BAC clones allowed positioning of bovine ZAR1 between BM4528 and BM4621 markers.

**Figure 2:**
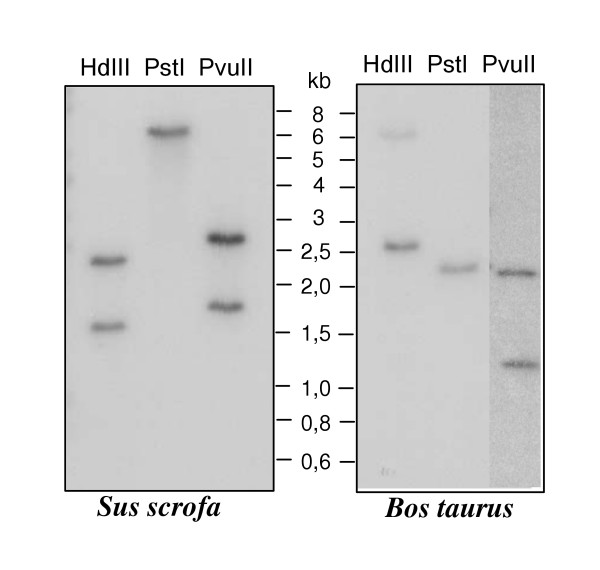
**Southern blot hybridization of porcine and bovine genomic DNA with ZAR1 partial cDNA probe. **Number of hybridized bands corresponded to predicted hybridization pattern of unique gene, fragmentized by HindIII (HdIII), PstI or PvuII endonucleases.

Southern-blot hybridization on *Sus scrofa *and *Bos taurus *genomic DNA revealed the presence of a single ZAR1 gene both in porcine and bovine genome (Fig. 2). As expected for one-copy gene, single band was detected in PstI-digested samples and 2 bands were hybridized when restriction by HindIII or PvuII endonucleases was performed in both species. These profiles corresponded to the patterns deduced from ZAR1 gene sequences analysis.

### Analysis of ZAR1 mRNA expression in different tissues, oocytes and preimplantation embryos in pig and cattle

**Figure 3:**
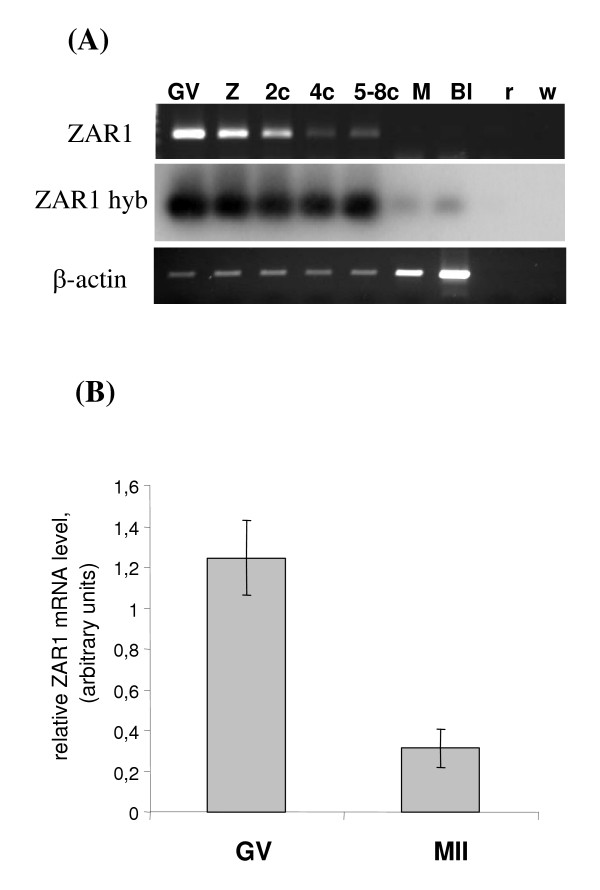
**ZAR1 expression in oocytes and preimplantation embryos.(A) **Expression ZAR1 mRNA in pig oocytes and embryos developed *in vivo*:GV – immature germinal vesicle oocyte, Z – presumptive zygote, 2c – 2 cells embryos, 4c – 4 cells embryos, 5-8c – 5–8 cells embryos, M – morulae, Bl – blastocyst, r – pool of embryos RNA RT omitted, w – RT with water instead of RNA. 35 and 30 PCR cycles were performed for ZAR1 and β- actin detection respectively. **(B) **ZAR1 mRNA expression in bovine oocytes before (GV, immature oocytes) and after *in vitro *maturation (MII, mature oocytes) quantified by real-time PCR. Data are presented as mean value of 3 experiments made in triplicate each +/- SEM. Difference is significant at p < 0.01.

By RT-PCR coupled with southern blot hybridization, level of polyadenylated ZAR1 mRNA was shown to decrease throughout pig embryo development from zygote to 8-cell stages, and only faint traces could be detected in morula and blastocyst even after radioactive hybridization of amplified products (Fig. 3A). In bovine in vitro produced embryos, we confirmed similar expression pattern, as previously shown [15], but by using another primer set S3-AS8 (fig. not shown). In addition, by real time RT-PCR, we showed that level of polyadenylated ZAR1 mRNA declined in bovine oocytes during in vitro maturation (Fig. 3B).

**Figure 4:**
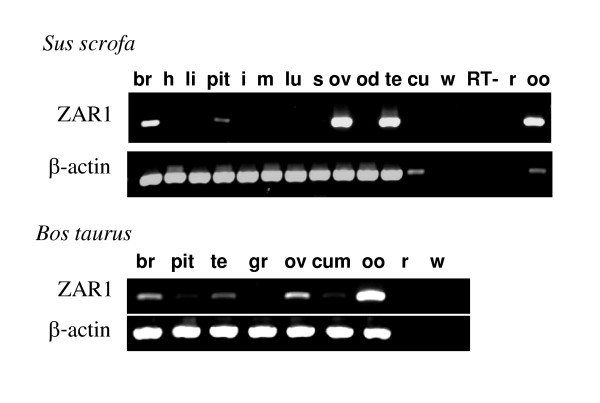
**Expression ZAR1 mRNA in porcine and bovine tissues. **Expression was detected by RT-PCR using S6-AS8 primers. 38 PCR cycles was performed for ZAR1, 32 and 38 cycles was performed to detect β-actin in porcine and bovine respectively. **br**- hypothalamus part of brain, **h **– heart, **li **– liver, **pit **– pituitary, **i **– intestine, **m **– muscle, **lu **– lung, **s **– spleen, **ov **– ovary, **od **– oviduct, **te **– testis, **cu **– cumulus cells, **gr **– granulosa cells, **oo **– oocyte, **w **– RT-PCR with water instead of RNA, **RT **– empty well, **r **– PCR with 1 μg of pooled RNA from tissues, RT omitted.

In both species, RT-PCR also revealed ZAR1 transcripts in hypothalamus, pituitary, ovary, testis, oocytes and occasionally in cumulus cells (Fig. 4).

### Different ZAR1 messengers in ovary and testis

**Figure 5:**
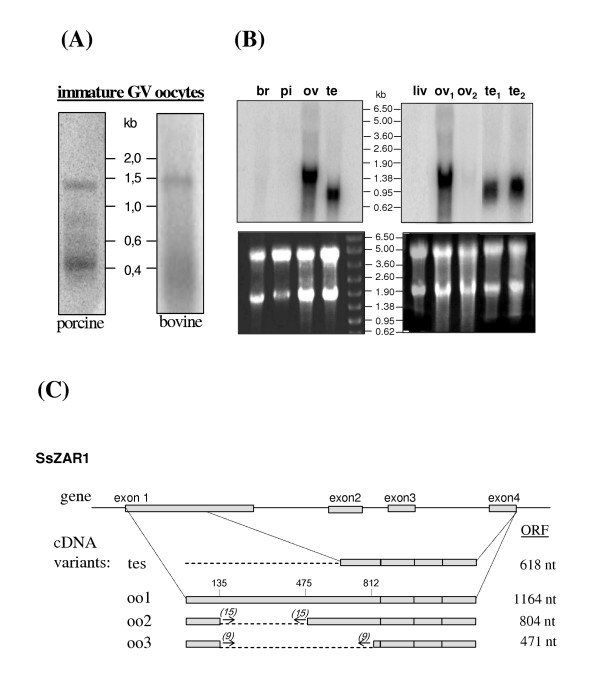
**Different ZAR1 mRNA variants. (A) **Detection of ZAR1 mRNAs in porcine and bovine oocytes by virtual Northern blot. Weight of molecular weight markers (kb) are indicated at the middle. **(B) **ZAR1 mRNA detection by classic Northern blot analysis in porcine tissues. Ethidium bromide RNA staining is at lower panels, hybridization is upper. Size of molecular weight markers (kb) are indicated in the middle. RNA from hypothalamic part of brain (br), pituitary (pi), ovaries (ov) of 7-week-old piglet and testis (te) of young boar were analysed (left gel). RNA from liver (liv), ovaries of other infantile immature (ov_1_) and peri-pubertal (ov_2_) gilts and testis of 7-month young (te_1_) and 2.5-year old (te_2_) boars were analysed. **(C) **Schematic representation of pig *ZAR1 *gene and correspondent cDNA variants, cloned from *Sus scrofa *oocytes (oo1, oo2, oo3) and testis (tes). The sizes of correspondent ORFs are indicated on the right. Grey bars designate exons. Chain line denoted sequences absent in tes, oo2 and oo3 cDNA variants. Palindrome repeats are indicated by arrows, their lengths are noted in brackets. The nucleotide coordinates of the beginning of palindrome repeats are marked relatively to oo1 cDNA sequence.

Virtual Northern blot on immature porcine and bovine oocytes revealed several ZAR1 transcripts of different sizes. The longest forms were about 1.4 – 1.5 kb in both species as expected according to obtained cDNA sequences (Fig. 5A). Lower bands and smear might correspond to some truncated transcript variants which we described above. Classic Northern blot on total RNA from different porcine tissues revealed major prominent transcript of approximately 1.7 kb in ovary and shorter transcripts of approximately 0.9 – 1.1 kb in testis (Fig. 5B). Faint bands of about 1.0 and 0.6 kb were detectable after longer autoradiography in ovary. The size of transcripts in pig ovary was in agreement with obtained cDNA sequences, it includes ORF, untranslated regions and 150–250 nucleotides poly-A tail. In ovaries from 7-month old peri-pubertal gilt ZAR1 mRNA was significantly less abundant than in 7 weeks of age piglet. No clearly visible messengers could be detected in porcine brain and pituitary as well as in liver RNA preparation used as negative control. In bovine, we failed to detect the prominent signal of ZAR1 transcripts in gonads or any other tissues of adult animals by this method.

Alignment of pig ZAR1 nucleotide sequences showed that cDNA variants, derived from oocyte, differed by deletions within the first exon, delimited by palindrome repeats (Fig. 5C). These repeats were 15 bp long sequences GGGGGCTACCCTCCT/AGCAGGGTAGCCCCC starting at nucleotide positions 135/475 in oo2 variant and 9 bp long repeats GGGGGCTAC/GTAGCCCCC at 135/812 nucleotides in oo3 form. Alignments of bovine oocyte ZAR1 transcript variants revealed numerous similar deletions within corresponding region (not shown).

To determine the origin of shorter ZAR1 messengers in pig testis, we performed 3'- and 5'-RACE and deduced the 802 nucleotides length sequence (GenBank accession DQ231445). It differed from full-length oocyte form by the absence of a part of the first exon at 5'-end (Fig. 5C). Testicular ZAR1 cDNA includes a 618 nucleotides long ORF which encodes putative 205 amino acid protein. This ORF starts from an alternative ATG codon at 547–549 nucleotide position relative to oocyte ZAR1 cDNA and has 100 % identity with its downstream sequence. Thus, different size transcripts in ovary and testis are likely to be transcribed from the same gene.

**Figure 6:**
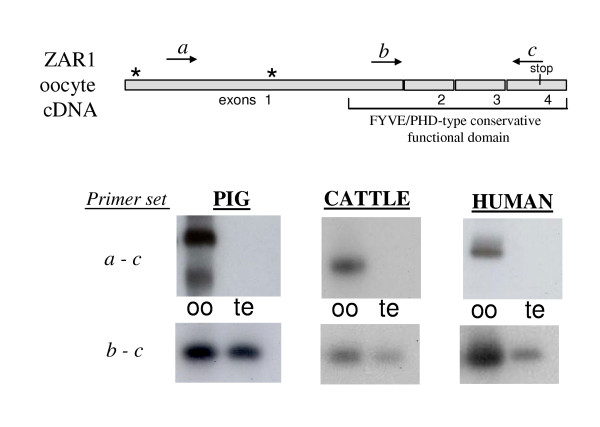
**RT-PCR detection of differently spliced ZAR1 transcripts in pig, cattle and human oocytes in comparison with testis. **Directions of primers situated on *a, b *and *c *positions are marked on ZAR1 cDNA scheme by arrows. Stars marked putative start codons, stop codon is noted. Specificity of amplified fragments was verified by southern blot hybridization using porcine full-length cloned ZAR1 cDNA as a probe.

Comparative RT-PCR analysis on oocyte and testicular full-length cDNA using different sets of primers were also performed in pig, cattle and human. When using *b-c *primers set, spanning exon 2–4, products of expected size were amplified in both oocyte and testis (Fig. 6). By contrast, if the sense primer was targeted to the sequence presumably absent in testicular transcript (set *a-c)*, PCR products were detected only in oocyte but not in testis in the three species (Fig. 6B). In human, as in porcine, methionin residues 173 and 183, respectively, could be used as alternative start ZAR1 sites in testis. In contrast, no additional methionin was found inside bovine full-length ZAR1 ORF (Fig. 1A).

### Localization of ZAR1 messengers in pig germinal cells by in situ hybridization

**Figure 7:**
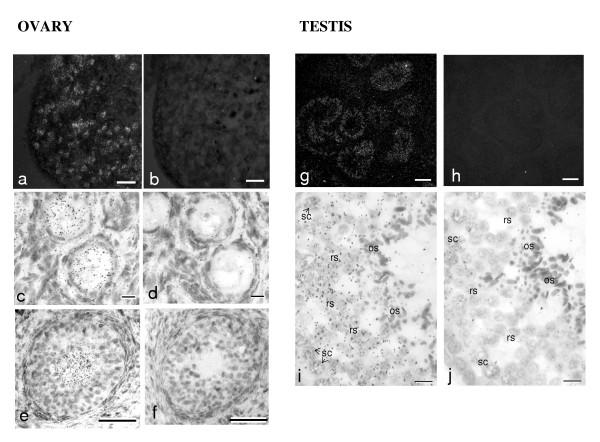
**Localization of ZAR1 mRNA in porcine ovaries and testis by in situ hybridization. **ISH was performed using specific ^35^S-labeled antisense (a, c, e, g, i) and control sense (b, d, f, h, j) probes. Dark-field images (a, g, bar 100 μm) showed ZAR1 expression within follicles in ovary and inside of seminiferous tubules in testis. It focused in oocytes of primary (c, bar 10 μM) and pre-antral follicles (e, bar 50 μM) and mainly in round spermatids (rs) but neither in oblong spermatids (os) no in primary spermatocytes (sc) – image i, bar 10 μM.

In immature pig ovary, expression of ZAR1 mRNA was restricted exclusively to oocytes both in primary and secondary pre-antral follicles and neither in granulosa cells nor outside the follicles (Fig 7A). In testis, ZAR1 was shown to be expressed only inside of seminiferous tubules but not in conjunctive tissues (Fig 7B). Specific RNA was detected in germinal cells, mostly in round spermatids and secondary spermatocytes. ZAR1 mRNA was neither expressed at significant level, if at all, in oblong spermatids, spermatozoa and primary spermatocytes, nor at the periphery of the tubules, containing spermatogonia and Sertoli cells bodies.

## Discussion

This study showed the high similarity of ZAR1 genes in pig and cattle as compared with human, as well as the common presence of FYVE/PHD zinc finger domain in deduced ZAR1 proteins. The FYVE ZnF domain is conserved from yeast to human. It functions in the membrane recruitment of cytosolic proteins by binding to phosphatidylinositol 3-phosphate [38]. The PHD domain is characteristic of transcriptional activators, repressors or cofactors. Taking into account these conserved protein functional domains in six vertebrate species, including human, mice, rat, xenopus, zebrafish and fugu, and expression patterns described in mice and frog oocytes and early embryos, ZAR1 may be considered as one of the transcriptional regulators acting during the oocyte-to-embryo transition of gene expression [19]. Reported here data supported this hypothesis but also supposed the possible ZAR1 involving in functioning of gonado-hypothalamic axis.

### Extra-oocyte ZAR1 mRNA expression in pig and cattle

By in situ hybridization we localised the ZAR1 messengers not only in growing oocytes, but also in testis, preferentially in secondary spermatocytes and round spermatids. Similar expression pattern was observed for an orphan nuclear receptor called germ cell nuclear factor (GCNF) since its expression was restricted to the post meiotic round spermatids and to the growing oocytes in mice [39]. GCNF acts as a repressor of gene transcription during preimplantation embryo development [40]. The possible role of ZAR1 in transcription repression in post-meiotic germ cells of pig and cattle might be supposed.

ZAR1 expression was not restricted to the gonads in pig and cattle but was also detectable in hypothalamus and pituitary. The members of recently discovered oocyte-specific gene family, T-cell leukemia/lymphoma 1B (*Tcl1b*) genes, are preferentially expressed in egg and early embryos, however correspondent ESTs were also found in testis and pituitary gland [41]. In mice also, expression of oocyte-specific *Bmp15 *was reported in gonadotrope cell line LbT2 and in pituitary. BMP15 was shown to be a potent and selective stimulator of FSH biosynthesis and secretion by the primary pituitary cells [42]. Reciprocally, the brain-derived neurotrophic factor (BDNF), initially identified to be an important regulator of neuronal survival and differentiation in bovine, has also been found in bovine oocyte and cumulus cells and may have a role in promoting oocyte cytoplasmic competence [43]. Among the ESTs, preferentially expressed in bovine oocyte, significant number was also detected in testis and brain [13]. We also found ESTs, coding for Zar1, in cDNA libraries from mouse brain (GenBank accessions BB248342, BF471866 and BE863668). Further investigations are required to determine the possible role of zygote arrest 1 in brain and pituitary in mammals.

ZAR1 expression was found by RT-PCR in frog muscle and lung, in addition to ovary [19]. Similarly, in cattle one study reported the amplification of a fragment homologous to ZAR1 in muscular cDNA (body and heart) [20]. In contrast, several studies failed to detect such expression in mice, human, pig and cattle [4], [15], this paper). This discrepancy could be explained by the fact that the 126 nucleotides cDNA fragment detected in that study [20] showed only 78% identity with ZAR1 bovine sequence reported here and may thus represent a part of a different gene with partial ZAR1 sequence homology. In addition, constant expression of this transcript throughout bovine preimplantation embryo development with sudden increase at 4-cell was reported [20], while summarizing data on ZAR1 expression in early embryos in pig (this study, [44]), cattle [15], mice and frog [4,19,1], it can be concluded that ZAR1 did not reactivate after maternal-embryo transition.

### Different ZAR1 mRNA variants in germ cells

Two types of ZAR1 mRNA variants were found. The first concerns the different size of ZAR1 mRNA in testis with regard to ovary, and could be the result of a differential splicing by shortening of 5'- end of the first exon in testicular messengers. Alternative splicing occurs in approximately half of all mammalian genes and its frequency is higher for tissue-specific rather than for ubiquitous genes [45]. Oocyte-specific *Mater *gene was reported to produce at least four differentially spliced mRNA variants in mice [46]. The cAMP-responsive element modulator (CREM) gene encodes a family of transcriptional regulators, which were generated by alternative splicing and alternative translational initiation in mice spermatids [47]. Difference of ZAR1 mRNA isoform in testis comparing to oocyte was confirmed in pig, cattle and human – in all three species testicular ZAR1 transcripts lacked a 5'-part of the first exon. In human and porcine, methionin residues, which could act as alternative translation initiation sites, were found. Although no downstream AUG codon was evidenced in bovine ZAR1, translation of a putative protein could be initiated from another codon, likely CUG at nucleotide position 517 or 526 (DQ231456). Mechanisms of alternative initiation of translation at non-AUG codon had not been clearly defined, but local RNA structure and particularly stable downstream hairpins could determine the non-AUG translation start site (for review -[48]). Numerous direct and inverted repeats, found within ZAR1 genes, might trigger such events. Interestingly, a band of about 35 kD was revealed by Western blot in addition to the 45 kD Zar1 in mice [4]. This might be the product of an alternative translation initiation site (Methionin at position 61). Indeed, the predicted molecular weight of such alternative protein (34 kD) corresponds to the western blot smaller band. The fact, that no smaller Zar1 mRNA variants were appreciated by Northern in mouse ovary could be explained by using the 1–180 nucleotides cDNA as a probe [4], whereas this fragment might be absent in shorter transcripts.

The second type of ZAR1 mRNA variants concerns ZAR1 isoforms that were found by RT-PCR in oocytes in both species. They included full-length cDNA, corresponding to what could be predicted from gene sequence, and several shorter variants, bearing relatively long deletions within the first exon. These deleted sequences were flanked by palindrome repeats, but were not in agreement with classical intron boundaries GT-AG or AT-AC rule (for review see [49]). Therefore, these cDNA variants could be the result of complex secondary structure of pig ZAR1 transcripts via stem loops forming and not really alternatively spliced forms. These loops, induced by the presence of palindrome repeats, could impair the generation of a full-length cDNA during RT. This could explain the origin of numerous lower bands and smears observed on virtual Northern blot, corresponding to truncated sequences.

### Elements of ZAR1 regulation

Numerous palindrome repeats were detected in highly GC-rich region within the first exon of ZAR1. Inverted palindrome repeats occur in prokaryotes and eukaryotes DNA and can form stem-loops and cruciform figures, which affect DNA structure or may interact directly with proteins [50]. Short palindrome repeats are characteristic of DNA-binding domain of nuclear receptors, but normally they are separated by only few nucleotides [51]. In mammals, palindrome repeats were reported mainly in 5'-upstream regions of several genes, such as mouse NF-kappa B [52] or human RNA polymerase II large subunit (RpII LS) encoding genes [53]. In the first case, these repeats were shown to be responsible for the induction of NF-kappa B by tumour necrosis factor (TNF-alpha), in the second example repeats lead to highly structured RpII LS RNA, which may be responsible for transcriptional regulation. This might be the case of ZAR1 genes, where repeated sequences within its coding region could be involved in their transcriptional regulation.

Two types of putative regulatory elements were determined within 3'-UTR of ZAR1 mRNA. The first one was a consensus UGUA XPum-bindind sequence [54], that we found in pig and cattle just before polyadenylation signal AAUAAA. We also found UGUA sequences at similar positions also in human at 1379–1382 nucleotides, in mice at 1210–1213 nucleotides, in rat at 1220–1223 nucleotides, and in *Xenopus laevis *ZAR1 mRNA at nucleotide position 1031–1034 (GenBank accessions NM_175619, BC099399, NM_181385, AY283176, respectively). In *Xenopus *oocytes, XPum protein (homologue of *Drosophila pumilio*) can physically bind to mRNA via UGUA sequence. Pumilio protein was shown to act as translational repressor of cyclin B1 by binding to this sequence during oocyte maturation, in addition to Cytoplasmic Polyadenylation Element (CPE) -mediated repression [55]. Pumilio proteins are highly conserved, bearing around 90% amino acid identity in vertebrates [55]. Interestingly, in bovine GV oocytes, the translation of cyclin B1 short mRNA isoform, lacking a CPE but bearing an UGUA sequence, was repressed [56]. Potentially, homologue of Pumilio protein could participate in the regulation of ZAR1 protein translation via UGUA.

The second putative regulatory sequences found within ZAR1 3'-UTR were stretches rich in U/purines dinucleotides repeats, sequence elements that are characteristic of embryo-deadenylation element (EDEN) in xenope maternal transcripts (c-mos, Eg5). They could drive rapid deadenylation and translational repression of these messengers in xenopus oocytes and embryos (for review [57]). Such regulation mechanism has not been reported in mammals yet. However, taking into account the similarity of polyadenylation mechanisms and mRNA translation via CPE-binding protein in xenope and mammals [58], we can speculate that as in xenopus, EDEN-like sequences could play a role in the regulation of expression of specific transcripts, including ZAR1, by deadenylation and further degradation, in oocyte and preimplantation embryos.

## Conclusion

Overall, porcine and bovine ZAR1 genes are highly homologous to human. Taking in account mRNA regulatory elements and differential expression patterns in germ cells through alternative splicing variants, ZAR1 might be considered as one of the regulator of post-meiotic events in germ cells in addition to its role in early embryo development. Species conservation of ZAR1 expression and regulation between human and domestic animals underlines the central role of this gene in early reproductive processes. Further studies are necessary to provide an insight in the role of this gene in functioning of gonado-hypothalamic axis.

## Authors' contributions

SU mainly designed this study, carried out the bovine cDNA cloning, experimental DNA and RNA analysis by blot hybridizations, RT-PCR and ISH, and drafted the manuscript. MRS performed cloning of cDNA variants in porcine. RDT participated in experimental work (bovine oocyte collection, RNA isolation and reverse-transcription), bioinformatic treatment of sequences and made substantial contributions to the analysis and interpretation of the data. CP collected the bovine oocytes and performed the in vitro production of preimplantation embryos. PP carried out the analysis by PCR and real-time PCR and was involved in DNA and RNA preparation. FM performed the regional assignment of studied gene by HRP and BAC screening. AT and SP were involved in performing RACE, Virtual Northern and ISH analysis and participated in study design. JC prepared the animals, collected the porcine tissues and helped in their analysis. VC and DR performed human oocytes collections and participated in manuscript preparation. PMo and PMe have been considerably involved in data interpretation and revising the manuscript critically for its content. All authors have read and approved the manuscript.

## Grant support

This work was sponsored by grants OVOGENAE from the French Ministry of Research (#03P409) and APISGENE.
